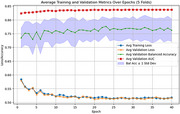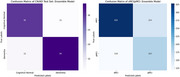# Cortical Graph Neural Networks to Predict Dementia Risk Based on MRI‐Derived Cortical Surface Morphonology

**DOI:** 10.1002/alz70856_107666

**Published:** 2026-01-09

**Authors:** Guanlin Guo, Harinishree Sathu, Marc D. Rudolph, Trey R. Bateman, Timothy M. Hughes, Suzanne Craft, Metin Nafi Gurcan, Sheng Luo, Da Ma

**Affiliations:** ^1^ University of Texas Health Science Center at Houston, Houston, TX, USA; ^2^ Duke University School of Medicine, Durham, NC, USA; ^3^ Georgia Institute of Technology, Atlanta, GA, USA; ^4^ Wake Forest University School of Medicine, Winston‐Salem, NC, USA

## Abstract

**Background:**

Alzheimer's Disease (AD) is the leading cause of dementia. Cortical atrophy patterns derived from T1 MRI are sensitive neuroimaging biomarkers to detect early signs of AD‐related neurodegeneration. However, early diagnosis at the prodromal stage is challenging due to the subtle and heterogeneous neuropathological and neurodegeneration patterns over the cortical surface, which cannot be captured by conventional deep learning methods natively. In this study, we developed an explainable cortical graph convolutional network (GCN) that captures the early signs of atrophy patterns in the cortical surface as graph‐based features to identify subjects with elevated AD risk.

**Method:**

T1 MRI data from the baseline visit of Alzheimer's Disease Neuroimaging Initiative (ADNI; 1645 subjects, 902 Male, 743 Female; stable NC [sNC]: 523, stable [AD]: 339, MCI: 783) dataset was processed through FreeSurfer (v7.4). Five‐fold cross‐validated models were built using 90% of the CN+AD data as training (stratified by gender and clinical diagnosis), with 10% reserved for independent testing. The dementia risk models were then applied to predict stable mild cognitive impaired (sMCI) vs. progressive MCI (pMCI) to evaluate their performance to predict future risk of dementia onset. A cortical GCN model handles the cortical surface mesh as graphic‐based input, where cortical morphological data (thickness and curvature) are defined on each vertex (graph node) and used as predictive features. During training, batch normalization and dropout were applied to each graph convolutional layer to mitigate overfitting and enhance training stability.

**Result:**

The cortical GCN classification model achieved a balanced accuracy of 0.736 in differentiating dementia (AD) and cognitively normal (CN) subjects and an average mean balanced accuracy of 0.644 for predicting sMCI from pMCI.

**Conclusion:**

This project demonstrated the effectiveness of using cortical GCN to achieve early prediction for future dementia onset for subjects with risk of AD. Future work will focus on independent validation of NACC/ADRC data to evaluate the model's generalizability, as well as enhance the model's explainability through techniques such as Grad‐CAM and integrated gradient. These efforts will provide deeper insights into AD's neuroanatomical aspects and contribute to developing more accurate, transparent, and effective diagnostic tools.